# Improving competencies in evidence-based dementia care: Results from a pilot study on a novel inter-professional training course (the KOMPIDEM project)

**DOI:** 10.3205/zma001034

**Published:** 2016-04-29

**Authors:** Katrin Balzer, Rhian Schröder, Anne Junghans, Ute Stahl, Jens-Martin Träder, Sascha Köpke

**Affiliations:** 1 University of Lübeck, Nursing Research Unit, Institute for Social Medicine and Epidemiology, Lübeck, Germany; 2 University of Lübeck, Medical Student, Lübeck, Germany; 3 Hamburg University of Applied Sciences, Faculty of Economics and Sociology, Department for Nursing and Management, Hamburg, Germany; 4 AMEOS Krankenhausgesellschaft Holstein mbH, Neustadt, Germany; 5 University of Lübeck, Institute for Family Medicine, Lübeck, Germany

**Keywords:** Dementia, Evidence-Based Practice, Education, Medical, Undergraduates, Education, Nursing, Diploma Programs, Inter-professional Relations, Problem-Based Learning

## Abstract

**Objective:** In order to address well-known challenges in dementia care, an inter-professional course was developed to improve medical students’ and nursing students’ competencies in the provision of evidence-based care for people with dementia. The course comprises lectures, problem-based learning (PbL) tutorials and visitations to care facilities. A pilot study was conducted to evaluate the acceptance and feasibility of the inter-professional course.

**Methodology:** Alongside preliminary implementation of the newly developed course, a pre-post survey was carried out involving all participating students. The questionnaire contained standardized and open-ended questions on participants’ views regarding the quality and relevance of several course components and characteristics. The data were analyzed by means of descriptive statistics.

**Results: **When the course was offered the first time, multiple barriers became evident, leading to premature course termination and subsequent revision of the curriculum. When the revised course was offered, 10 medical students and 8 nursing students participated. The course proved feasible, and the median overall quality was rated as “2” (IQR 2–3) at a rating scale ranging from 1 (very good) to 6 (inadequate). Following aspects were most frequently judged positively: the course’s inter-professional scope, the visitations to care facilities and the PbL tutorials. Potential for improvement was particularly noted with regard to a more distinct focus on well-defined, mainly practical learning outcomes. There were no indications of systematic between-group differences in the medical and nursing students’ perceptions of the course program.

**Conclusions: **The results confirmed the feasibility of the inter-professional course on dementia care and the relevance of its inter-professional scope. However, to ensure sustainable course implementation in the long term, further program adaptations based on current findings and further evaluation studies focusing on objective parameters of the process and outcome quality are required.

## Zusammenfassung

**Zielsetzung:** Um bekannte Herausforderungen in der Versorgungspraxis zu adressieren, wurde eine interprofessionelle Lehreinheit zur Verbesserung der Kompetenzen von Medizinstudierenden und Pflegeauszubildenden für eine evidenzbasierte Versorgung von Menschen mit Demenz entwickelt. Im Rahmen einer Pilotuntersuchung wurden die Akzeptanz und die Machbarkeit der aus Vorlesungen, problemorientiertem Lernen (POL) und Hospitationen in Pflegeeinrichtungen bestehenden Lehreinheit untersucht.

**Methodik: **Begleitend zur Pilotierung der Lehreinheit erfolgte eine Prä-Post-Befragung der Teilnehmenden. Der Fragebogen enthielt standardisierte und offene Fragen zur Bewertung der Lehreinheit aus der Sicht der Teilnehmenden. Die Daten wurden deskriptiv statistisch ausgewertet.

**Ergebnisse: **Bei der ersten Erprobung wurden mehrere Barrieren deutlich, die einen vorzeitigen Abbruch und eine Überarbeitung des Konzepts erforderlich machten. An der überarbeiteten Lehreinheit nahmen 10 Medizinstudierende und 8 Pflegeauszubildende teil. Die Lehreinheit erwies sich als durchführbar und wurde im Median mit der Schulnote 2 (IQR 2–3) bewertet. Am häufigsten uneingeschränkt positiv beurteilt wurden das interprofessionelle Lernen, die Hospitationen und das POL. Verbesserungspotenzial zeigte sich in der Ziel- und Praxisorientierung der Lehreinheit. Es fanden sich keine Hinweise auf systematische Unterschiede zwischen den beiden Lernendengruppen in der Wahrnehmung der Lehreinheit.

**Schlussfolgerungen:** Die Ergebnisse bestätigen die Machbarkeit der Lehreinheit und die Relevanz der interprofessionellen Ausrichtung. Für die längerfristige Verstetigung des Lehrangebots sollten jedoch weitere Anpassungen auf der Basis dieser Ergebnisse sowie objektivierende Evaluationen der Prozess- und Ergebnisqualität erfolgen.

## 1. Introduction

Dementia is a clinical syndrome which predominantly arises in old age due to different aetiologies, and is characterized by the progressive loss of cognitive functions [1]. According to epidemiological estimates, the prevalence of the illness amounts to roughly 10% within the age group of those over 65 years in Germany, with the annual incidence being just about 2% [2]. 

The care for and treatment of people with dementia entails substantial challenges for all persons involved, not just because of the affected persons’ cognitive decline, but also because of the non-cognitive or neuropsychiatric symptoms which arise over the course of the illness, such as restlessness, aggression and depression [3], [4], [5]. National as well as international empirical findings indicate that medical and nursing care for those affected by neuropsychiatric symptoms is often not delivered in accordance with current evidence-based recommendations [6], [7], [8]. Besides deficits in professionals’ knowledge and competencies [3], [9], [10], problems related to the cooperation between physicians and nurses have found to play a role [11], [12], [13]. This stands in distinct contrast with the much-emphasized necessity of continuous, effective collaboration between these two professions in the care for people with dementia [11], [14]. 

Reported shortcomings suggest that physicians and nurses are insufficiently prepared by their professional education to care for people with dementia. In Germany, dementia is subject both to medical and nursing education; however, respective lessons vary in type and scope across medical faculties and nursing schools, with uncertain impacts on the acquisition of required competencies [15], [16], [17]. Evaluation findings from other countries indicate that additional courses in medical and nursing education can induce positive changes in future healthcare professionals’ knowledge, attitudes and confidence regarding dementia care [18]. While all evaluated training programs differ in scope, content, methodical-didactic aspects and intensity, they consistently include a kind of practice-oriented course component. Subject to methodical limitations of existing research evidence, evaluation results furthermore indicate that the intended achievement of knowledge and attitude changes may be facilitated by introductory theoretical lessons on dementia and pre-existing practical experiences of the students in the general care for ill people. 

However, the investigated training programs exclusively target single professions, e.g. future physicians and nurses [18]. This negates potential cross-professional overlaps in required competencies, as well as the importance of effective physician-nurse cooperation in the care of people with dementia. Collective inter-professional learning arrangements, integrated in undergraduate training, would offer the opportunity to promote reciprocal understanding between (future) physicians and nurses early on in the professional careers [19], [20]. But to reach these effects, dementia care training programs are needed that recognise variation in the scope and education level (e.g. academic versus non-academic levels) of involved degree programs, e.g. medical and nursing degree programs. However, when our project was launched, courses meeting the particular requirements of medical education (academic level) and nursing education (mostly non-academic level) in Germany could not be identified in the literature. 

## 2. Objective

The objective of this project was to develop and pilot an inter-professional undergraduate training course on dementia care which has the potential to enhance medical and nursing students’ confidence in their competencies to deliver joint evidence-based care for people with dementia, irrespective of the care setting in question. The desired course was challenged to ensure effective collective learning processes despite existing differences in the levels of involved education programs (academic medical education and non-academic vocational training in nursing), the students’ prior practical experiences and their learning needs and conditions.

## 3. Project Course and Methodology

The project “Bessere KOMpetenzen für die interProfessionelle und Individuell angemessene Versorgung von Menschen mit DEMenz [Better COMpetencies for the inter-professional and Individually appropriate care for people with DEMentia] (KOMPIDEM)” was carried out at the University of Lübeck from October 2013 to February 2015. Institutions participating in the project were the Nursing Research Unit at the Institute for Social Medicine and Epidemiology, University of Lübeck, the Institute for Family Medicine, University of Lübeck, as well as the Nursing School at the Academy of the University Hospital of Schleswig-Holstein (UKSH Academy). The project comprised two stages: the development of the course curriculum, followed by its piloting and explorative evaluation. The present paper mainly focuses on the second stage.

### 3.1. Development and description of the training course

Right at the beginning of the project, the project members agreed on that the course program should primarily address medical students at the beginning of their clinical training phase (semesters 5 to 7 out of 12 semesters study program) and general nursing students in their third year of training (semester 5 out of 6 semesters vocational training program) with a specialization in geriatric care. However, the focus on medical students in their clinical training phase was not set as a rigorous limitation; basically it was agreed on that the training program should be available to all medical students who were interested.

For the development of the inter-professional course program a literature analysis and four focus group interviews with medical students and nursing students (specializing in geriatric care) were conducted (see Table 1 (Tab. 1)). Based on these findings, three major topics relevant to the target groups were identified:

1. Causes of cognitive and non-cognitive symptoms of dementia,
2. Strategies for successful communication with affected persons and
3. Strategies for appropriate treatment of cognitive and non-cognitive disease symptoms.

With regard to the teaching formats and methods, a combination of theoretical lessons and practically orientated course formats was found to be advantageous. 

**Figure 1:**
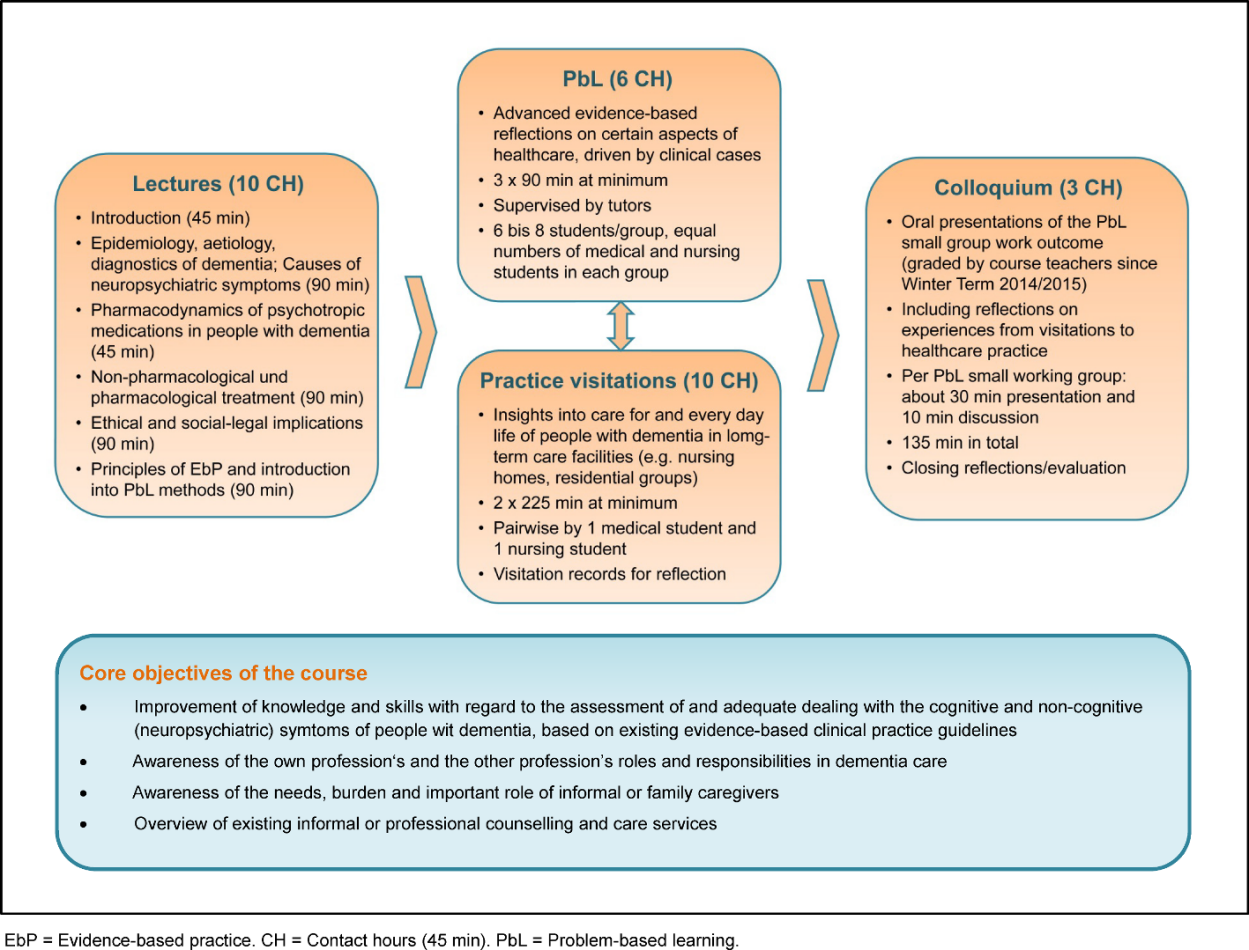
Core objectives and components of the course

Guided by these results, the learning objectives, the content and the methodological characteristics of the course program were defined (see Figure 1 (Fig. 1)). The course consisted of four components:

1. Introductory lectures to ensure a common basic knowledge level among all participants,
2. Problem-based learning (PbL) in small groups to promote a more detailed and activity-oriented engagement of the students with certain aspects of dementia care as well as inter-professional communication,
3. Visitations to care facilities to promote students’ awareness of existing challenges and problem-solving strategies applied in the care for people with dementia, and
4. A final colloquium comprising presentations by the PbL groups.

The contact hours of the participants with lecturers, tutors or healthcare representatives amounted to about 30 hours in total (equalling to one credit point). 

The course program was integrated as an optional course into the medical degree program of the University of Lübeck. Within the general nursing training at the UKSH Academy Lübeck, the program became an obligatory component of the third-year specialization in geriatric care, usually taken up by six to eight students. We aimed to enrol 20 participants, with an equal proportion of medical and nursing student if possible.

To raise medical students’ awareness of the new training program, the course was included in the optional subject catalogue of the University’s medical degree program. Additionally, multiple emails were sent to students currently enrolled in this degree program. General nursing students with a specialization in geriatric care were informed multiple times in person about the objectives and structure of the training program. Furthermore, the course teacher was informed about this new training program.

#### 3.2. Piloting 

The objective of the piloting stage was to explore the acceptance and feasibility of the newly developed training course and to identify suitable instruments for the measurement of changes in participants’ competencies. For the piloting, it was planned to implement the course on a one time basis in the Summer Term of 2014, including a pre-post-survey with the participants. During the first piloting, however, a need for a second implementation and evaluation in the Winter Term 2014/15 became manifest. 

##### 3.2.1. Study participants

The target group of the survey was all medical and general nursing students participating in the training course. At the first lecture, they were orally informed about the objective and procedures of the survey. In addition, written information about the study objective, the data management and the data protection measures were included in the questionnaire. By completing the questionnaire, the student consented with participation in this study. The survey was carried out anonymously. To allow intra-individual mapping of the questionnaires, the participants were asked to sign all questionnaires with an algorithm-driven individual code only known to them. The intra-individual mapping was required for paired group comparisons as part of the integrated psychometric investigations.

##### 3.3.2. Data collection

**Figure 2:**
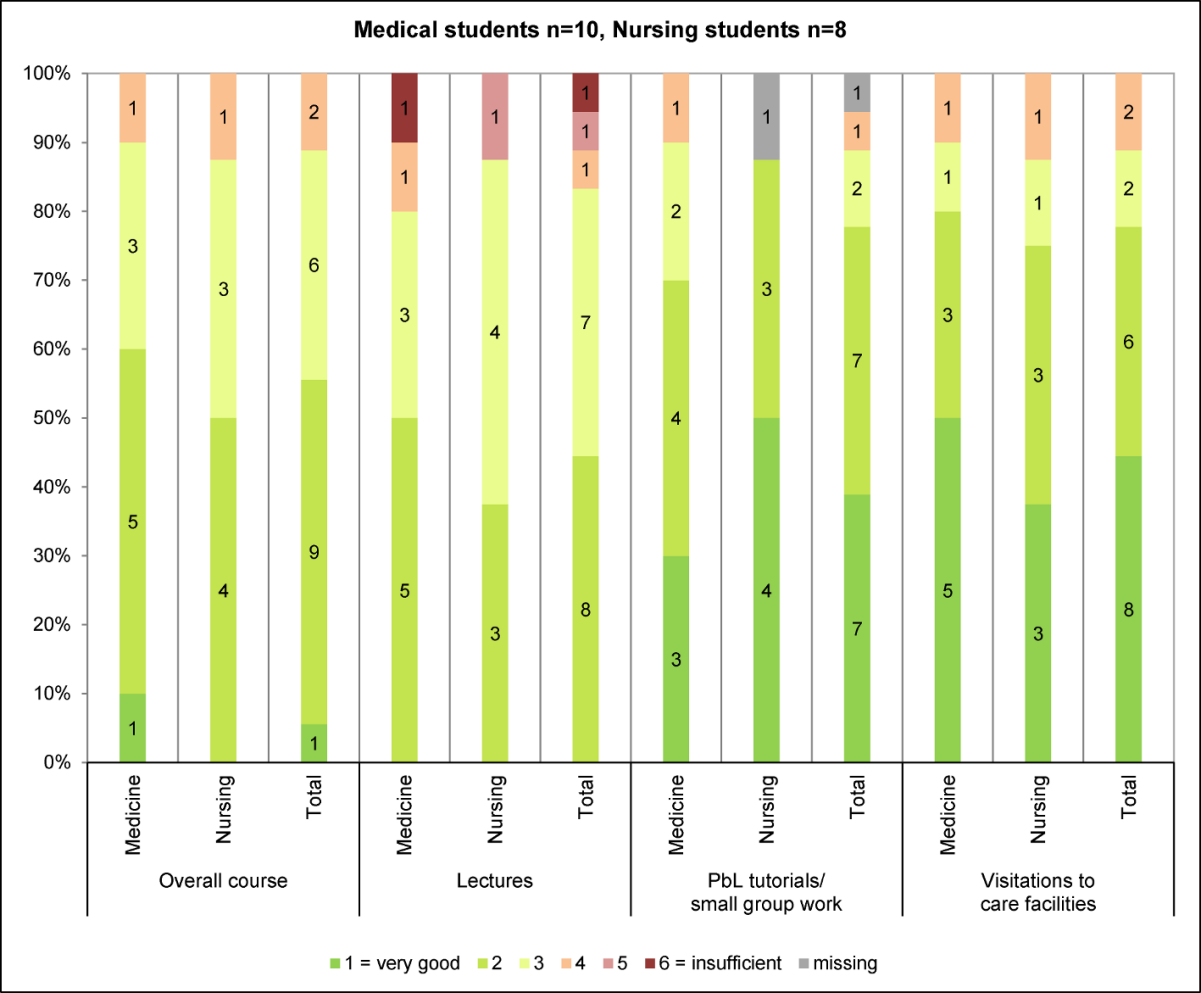
Overall assessment of the course curriculum and its single components [21]

**Figure 3:**
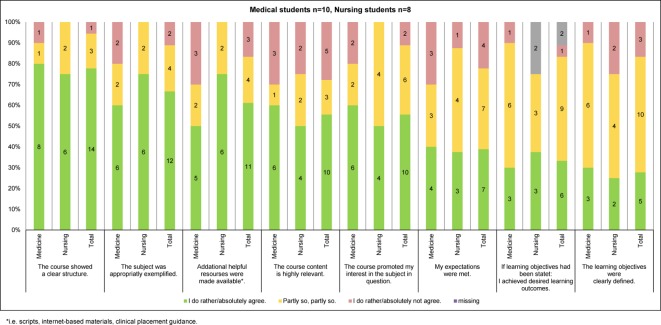
Evaluation of individual course aspects [21]

A written questionnaire was filled in by the participants at the beginning (T0) and the end of the course (T1). The questionnaires covered following aspects: socio-demographic details (T0), expectations on the course program (T0), subjective assessment of the course quality and several course characteristics (T1), and participants’ knowledge and attitudes regarding different aspects of the care for people with dementia (T0 and T1). For the survey of participants’ knowledge and attitudes, mainly instruments were used which had been newly developed for the purpose of this study or had not yet been tested in the study population of interest. Respective survey results were only used for psychometric evaluation of these instruments and will be reported elsewhere together with findings from still ongoing psychometric evaluation studies. For the subjective assessment of the course quality and other course characteristics, a modified version of Zumbach’s short scale on teaching evaluation [21] was used. It was combined with further standardized and open-ended questions specifically designed for this study, among them items on the course’s inter-professional scope (see Figure 2 (Fig. 2) and Figure 3 (Fig. 3), Table 2 (Tab. 2)).

The questionnaires were handed out to the students at the beginning of the first course session and at the end of the last course session, and filled in directly in the presence of a member of the project team. At each measuring point, completion of the questionnaire took 30 minutes on average.

##### 3.3.3. Data analysis

The quantitative data was analyzed descriptively (IBM SPSS Statistics 22, Microsoft^®^ Excel 2010). For interval-scaled variables, the median and the interquartile range (IQR) were determined, for ordinal-scaled or categorical variables the proportions per scale step or answer category were computed. All analyses were carried out for the total sample and stratified for the sub-samples of medical and nursing students. The answers to the open-ended questions were content analyzed. 

## 4. Results

### 4.1. Piloting in Summer Term 2014

Six general nursing students with a specialization in geriatric care as well as two medical students (4th Semester and 10th Semester) signed up for the course in the Summer Term 2014. While further medical students enrolled in the target semesters 5-7, in particular those participating in the preceding focus groups, voiced an interest in the course, they were not able to take part due to overlaps with compulsory classes. Timing the course in another way was not possible as otherwise interferences would have been occurred with the nursing degree program’s time table. 

Over time, great reservations occurred among participating nursing students regarding the new course. The following concerns were mentioned:

1. Low participation rate of medical students,
2. Obligatory course participation for nursing students compared to voluntary course participation for medical students,
3. Concerns that the course would overlap too much with other courses within the nursing degree program or expected low benefits for the learning outcome,
4. Perceived difficulties in the organization and coordination of course appointments especially during clinical placement intervals and
5. The course perceived as being too time-consuming (e.g. due to travel times, self-directed learning).

Despite multiple discussions with the students themselves, the course teacher and other students’ mentors, the students could not be convinced to continue participation, leading to premature course termination before the PbL tutorials and visitations to care facilities began. 

Data from these participants was exclusively collected at T0. Due to the lacking T1 measurements, the T0 will not presented here. 

#### 4.2. Piloting in Winter Term 2014/15

In response to the first piloting experiences, the curriculum was revised and the course was offered once more in the Winter Term 2014/15. The introductory lectures were combined to a compact 1.5 day course, and it was decided to refrain from the compulsory character of the course for nursing students specialising in geriatric care. Instead, the course was now offered as a voluntary class to all third year nursing students regardless of the chosen specialization, including those specializing in paediatric nursing care. To make the optional course more widely known, it was presented in person during one compulsory lecture for medical students enrolled in the target semester 5, and 500 leaflets were distributed at frequently visited campus places at the beginning of the Winter Term 2014/15.

##### 4.2.1. Participants

In the Winter Term 2014/2015, 10 medical students (n=4 semester 5, n=5 semester 7, n=1 missing data) and 9 out of 24 third year nursing students signed up for the inter-professional course on dementia care. One nursing student had to withdraw his participation due to sick leave, leaving a total sample of 10 medical students and 8 nursing trainees (5 general nursing students and 3 paediatric nursing students) participating in the course. They all completed the pre-post survey. 

Table 2 (Tab. 2) summarises the sample characteristics. In both sub-samples, communication with people with dementia was among the course subjects most frequently expected by the students. With regard to the students’ further expectations on the course content, some differences between the two sub-samples were noted. In each sub-sample, half of the participants mentioned the inter-professional course scope as being particularly appealing to them.

#### 4.2.2. Feasibility and program quality

All course components could be carried out as planned. All participants attended all lectures and scheduled visitations to the care facilities; the average number of attended PbL sessions amounted to 2.8 per participant (expected number was 3 per participant). Non-attendance affected two medical students (n=1 attending 2/3 classes, n=1 attending 1/3 classes) and was justified by sick leave (1 missed session) or concurrent compulsory lectures (2 missed sessions).

Both sub-samples rated the course as a whole with a grade of “2” (IQR 2-3) on average. Of the individual course components, the PbL tutorials and the visitations to the care facilities were most frequently rated with the grades 1 or 2 (see Figure 2 (Fig. 2)). The experience of inter-professional learning was almost exclusively judged positively, with unlimited positive judgements being slightly more frequently made by the nursing students compared to the medical students (see Table 3 (Tab. 3)). The provided amount of course content and the pace of teaching were found to be appropriate by just about half of the students, with slight differences between the two sub-samples (see Table 3 (Tab. 3)). The results on the remaining course characteristics are summarized in Figure 3 (Fig. 3). Some of them also show slight but inconsistent differences between the medical students’ and the nursing students’ ratings. The aspects least frequently rated positively by each of the two sub-samples were the clarity of learning objectives as well as the subjectively perceived learning achievements. In both sub-samples, less than half agreed fully or to some degree on the statement that their expectations had been met. Likewise, all participants named subject areas that should have been addressed in more detail, with non-pharmacological interventions and dealing with neuropsychiatric symptoms being mentioned most frequently (see Table 3 (Tab. 3)). 

Participants’ answers to open-ended questions (see Table 3 (Tab. 3)) correspond with the answers on the standardized questions and, furthermore, point to potential curriculum adjustments (e.g. with regard to a stronger practical orientation) and likely barriers (e.g. due to difficulties in the PbL tutorials or time overlaps with compulsory courses). 

## 5. Discussion

The piloting of the revised curriculum showed that the newly developed inter-professional course on dementia care is feasible in its current shape and can reach the desired participation rate among targeted medical and nursing students. Furthermore, students’ feedback confirmed the particular value of the inter-professional course scope. 

Of the individual course components, the visitations to care facilities were perceived as positive above all – both by the medical and the nursing students. This corresponds to the distinct interests of both sub-samples in the acquisition of practically oriented competencies with regard to the communication with people affected by dementia and the treatment of neuropsychiatric symptoms. However, the participants’ judgements also revealed that, despite the visitation component, the course program was only partially perceived as being effective in the promotion of these competencies. This finding is confirmed by a recent synthesis of evidence which points to a need for more effective strategies for the training of undergraduate healthcare students’ competencies in the communication with and person-centred care for people with dementia [18].To improve undergraduate education in this respect, training strategies proven effective in continuing professional education to improve the quality of dementia care should be taken into account [22]. Concerning the current course program in question, also a critical review of the scope and amount of each course component, i.e. lectures, PbL tutorials and visitations to care facilities, is required. Training strategies have to be identified which allow more effective dealing with the variety of participants’ expectations on the course and their heterogeneous levels of pre-existing knowledge and practical experiences, depending on their professional background (medical or nursing students) and the years of studying (early versus advanced students).

The PbL tutorials were mainly, but not consistently perceived as a positive experience. Existing differences in the judgements may be determined by various factors, among them individual learning strategies [23] or the tutor-students interplay [24]. The present study provides only limited insights into the type and cause of negatively connoted perceptions. Free-text answers suggest that the effectiveness of the PbL tutorials may have been hampered by uncertainties among the students regarding the expected learning outcomes and differences between medical and nursing students in the literature searching skills. Thus, for revision of the PbL component, existing theoretical and empirical evidence on PbL [25], [26], [27] should be scrutinised in order to identify PbL methods proven effective for student groups with pronounced skill mixes. 

The descriptive results of this pilot study does not provide any indication of systematic differences between the medical and the nursing students in the perception of the newly developed inter-professional training course, apart from slightly divergent judgements on some aspects. While the relevance of these differences cannot be reliably assessed based on current data, it has to be stressed that both sub-samples unanimously judged the clarity of targeted learning outcomes and the subjectively perceived learning achievements least positively compared to other course aspects. To improve students’ satisfaction in this respect, the target competencies of the training course should be revised. They should reflect more distinctly the learning needs and interests frequently mentioned by both the medical and the nursing students and should comply with recently recommended competencies frameworks for these target groups [28], [29], [30]. 

Although the course curriculum proved itself feasible in the second piloting, potential barriers became obvious. First of all, these relate to the limited compatibility of the course schedule with the medical students’ time tables of compulsory classes and the timeline of the nursing students which significantly differs from that of the medical students due the non-academic level of the nursing degree program. While full compatibility may be difficult to be reached within given boundaries, the course schedule should be reconsidered in order to identify further potential to improve its compatibility with involved undergraduate programs. Furthermore, it has to be kept in mind that the achieved participant rate of 8 nursing and 10 medical students was preceded by numerous awareness raising activities. To ensure that the optional course will remain well accepted in future, it is important that it will consistently be implemented at high levels of process and outcome quality, thus demonstrating its relevance and benefits. 

## 6. Limitations

Interpretation of the findings has to take into account the small sample size and purely descriptive nature of this study. Furthermore, the study data solely reflect the participants’ subjective perception. For a more conclusive evaluation of the course program, the learning processes, e.g. the self-directed learning, as well as the perspectives of other persons involved, e.g. teachers and representatives of visited care facilities, should be subject to research, too. Also, results from objective measurements of the learning outcomes by means of validated instruments have to be awaited. For the interpretation of the current findings it also has to be considered that the pilot study was planned and carried out by members of the project team (KB, AJ, SK) who additionally acted as PbL tutors within the course. While this involvement in the program delivery entails a certain risk of bias towards socially desirable answers, the range of critical feedback received from the participants indicates that it rather not affected the study findings.

## 7. Conclusions

Supported by the confirmed feasibility, the newly developed inter-professional course on evidence-based dementia care will be regularly offered as an optional class to medical students at the University of Lübeck and nursing students at the UKSH Academy Lübeck. To ensure the course’s long-term acceptance, feasibility and effectiveness, a further revision of the curriculum based on current findings is regarded as required. As foundation for this revision, a model of target competencies for dementia care should be developed, which details the competencies equally relevant to future physicians and nurses and takes into account the students’ learning needs as derived from this study as well as evidence-based recommendations for dementia care. With such a model, better transparency of the desired learning objectives and a more pronounced focus on the competencies essentially required in clinical practice, e.g. with regard to the treatment of neuropsychiatric symptoms, may be achieved. 

The next times the course will be delivered, systematic inquiries of the process and outcome quality should be included. Driven by the current findings, further studies are currently underway in order to develop and test instruments for the competency-based measurement of learning outcomes relevant to inter-professional dementia care. 

## Acknowledgements

We would like to thank all the students who participated in the course and the survey and thus significantly contributed to this study. We also wish to express our gratitude to all care facilities and teachers involved in the course implementation.

## Funding

The KOMPIDEM Project was funded by the Robert Bosch Foundation (Grant No.: 32.5.1316.0007.0).

## Competing interests

The authors declare that they have no competing interests.

## Figures and Tables

**Table 1:**
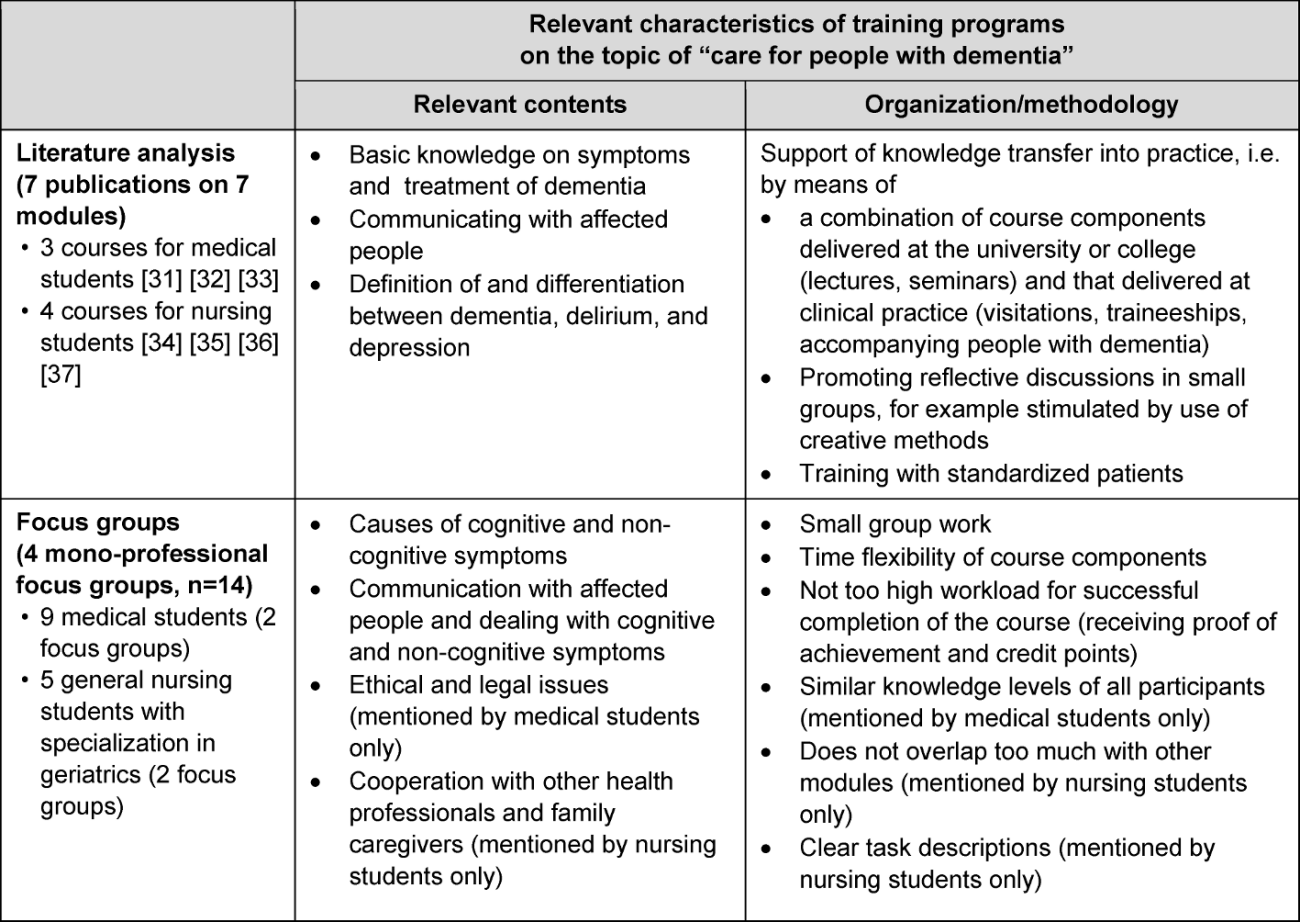
Table1: Results of the preceding literature analysis and focus groups

**Table 2:**
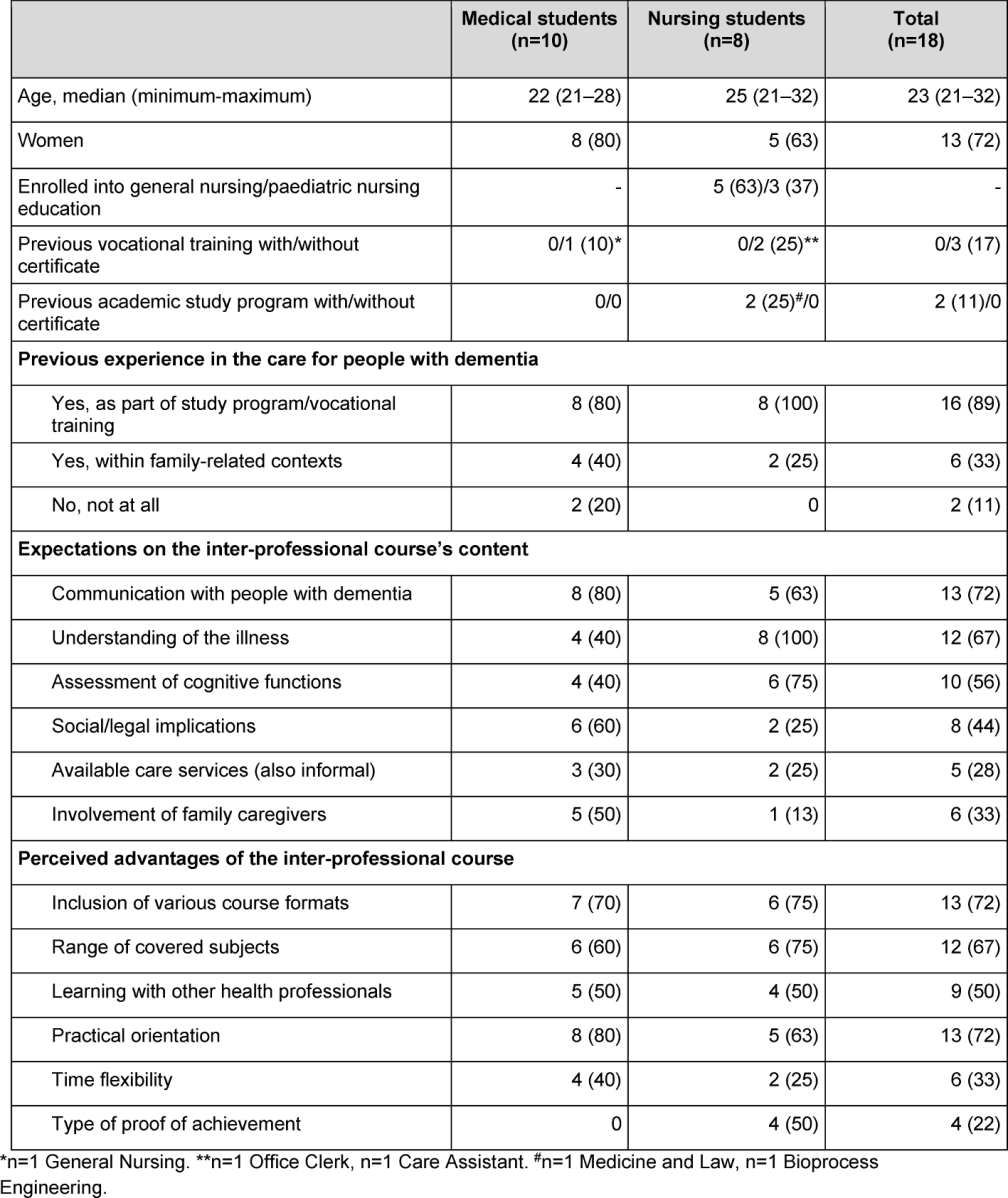
Sample characteristics (n (%) if not indicated otherwise)

**Table 3:**
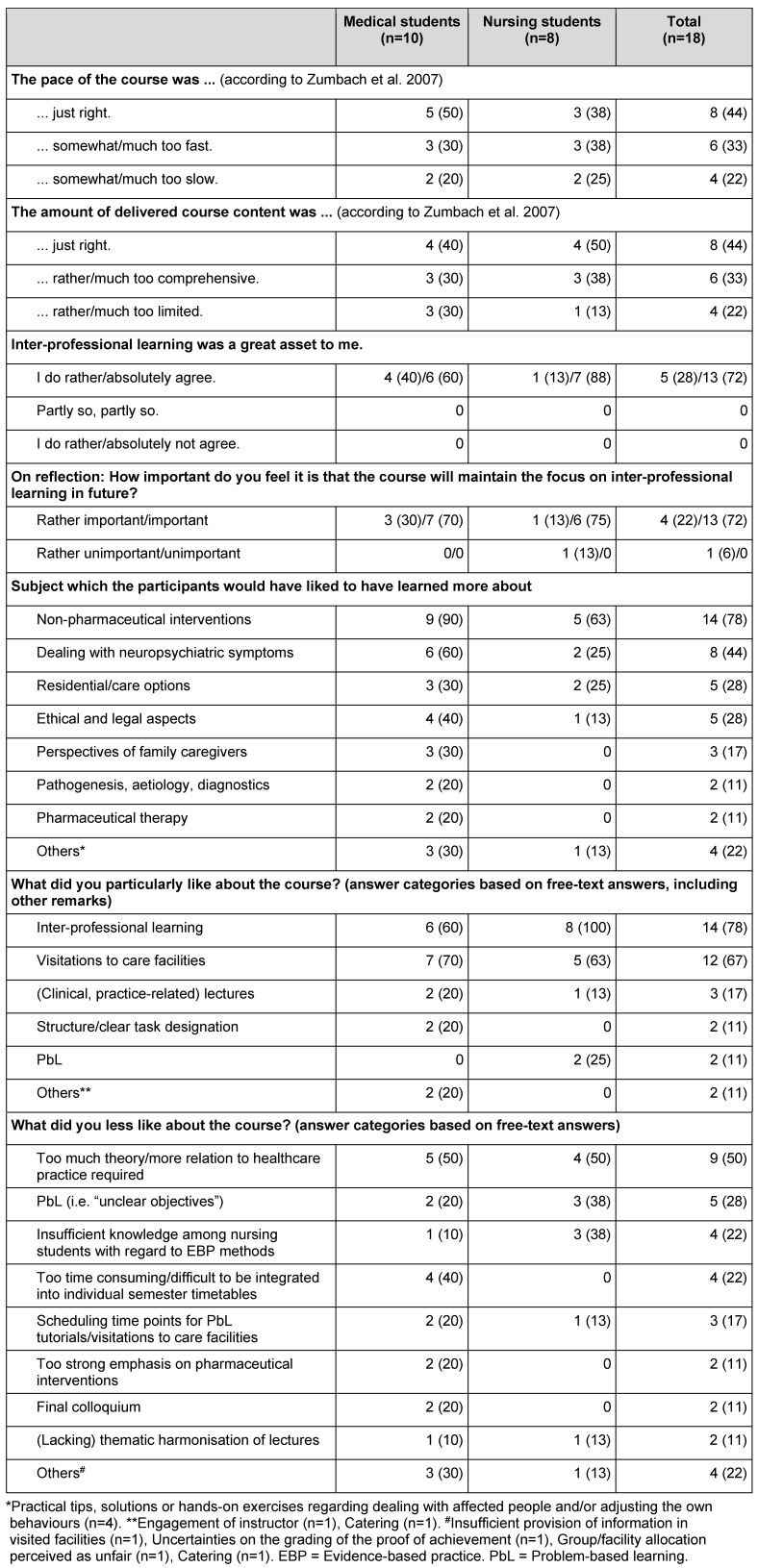
Evaluation of different aspects of the course program (n (%) if not indicated otherwise)

## References

[1] Kopf PD, Rösler A (2013). Demenz. Internist.

[2] Bickel H (2014). Die Häufigkeit von Demenzerkrankungen.

[3] Balzer K, Junghans A, Brinkmeier K, Jacobs B, Köpke S (2013). Aus Barrieren Chancen machen. Pflege von Patienten mit Demenz im Akutkrankenhaus. Pflege Z.

[4] Kales HC, Gitlin LN, Lyketsos CG (2015). Assessment and management of behavioral and psychological symptoms of dementia. BMJ.

[5] Teipel SJ, Thyrian JR, Hertel J, Eichler T, Wucherer D, Michalowsky B, Kilimann I, Hoffmann W (2015). Neuropsychiatric symptoms in people screened positive for dementia in primary care. Int Psychogeriatr.

[6] Balzer K, Butz S, Bentzel J, Boulkhemair D, Lühmann D (2013). Beschreibung und Bewertung der fachärztlichen Versorgung von Pflegeheimbewohnern in Deutschland.

[7] Köpke S, Mühlhauser I, Gerlach A, Haut A, Haastert B, Möhler R, Meyer G (2012). Effect of a guideline-based multicomponent intervention on use of physical restraints in nursing homes: a randomized controlled trial. JAMA.

[8] Sköldunger A, Fastbom J, Wimo A, Fratiglioni L, Johnell K (2015). Impact of Inappropriate Drug Use on Hospitalizations, Mortality, and Costs in Older Persons and Persons with Dementia: Findings from the SNAC Study. Drugs Aging.

[9] Cohen-Mansfield J, Jensen B, Resnick B, Norris M (2012). Knowledge of and attitudes toward nonpharmacological interventions for treatment of behavior symptoms associated with dementia: a comparison of physicians, psychologists, and nurse practitioners. Gerontologist.

[10] Pentzek M, Abholz HH, Ostapczuk M, Altiner A, Wollny A, Fuchs A (2009). Dementia knowledge among general practitioners: first results and psychometric properties of a new instrument. Int Psychogeriatr.

[11] van den Bussche H, Jahncke-Latteck ÄD, Ernst A, Tetzlaff B, Wiese B, Schramm U (2013). Zufriedene Hausärzte und kritische Pflegende – Probleme der interprofessionellen Zusammenarbeit in der Versorgung zu Hause lebender Menschen mit Demenz. Gesundheitswesen.

[12] Schaeffer D, Wingenfeld K (2008). Qualität der Versorgung Demenzkranker: Strukturelle Probleme und Herausforderungen. Pflege Gesellschaft.

[13] Smeets CH, Smalbrugge M, Zuidema SU, Derksen E, de Vries E, van der Spek K, Koopmans RT, Gerritsen DL (2014). Factors related to psychotropic drug prescription for neuropsychiatric symptoms in nursing home residents with dementia. J Am Med Dir Assoc.

[14] Bryon E, Gastmans C, de Casterlé BD (2012). Nurse-physician communication concerning artificial nutrition or hydration (ANH) in patients with dementia: a qualitative study. J Clin Nurs.

[15] Görres S, Stöver M, Bomball J, Schwanke A (2011). Demenzsensible nicht medikamentöse Konzepte in Pflegeschulen. Vermittlung pflegerischer Kompetenzen in der Ausbildung, die zur nachhaltigen Verbesserung von Menschen mit Demenz in Akutkliniken beitragen.

[16] Hasselbalch SG, Baloyannis S, Denislic M, Dubois B, Oertel W, Rossor M, Tsiskaridze A, Waldemar G (2007). Education and training of European neurologists in dementia. Eur J Neurol.

[17] Tsolaki M, Papaliagkas V, Anogianakis G, Bernabei R, Emre M, Frolich L, Visser PJ, Michel JP, Pirttila T, Olde Rikkert M, Soininen H, Sobow T, Vellas B, Verhey F, Winblad B, European Alzheimer Disease Consortium (2010). Consensus statement on dementia education and training in Europe. J Nutr Health Aging.

[18] Alushi L, Hammond JA, Wood JH (2015). Evaluation of dementia education programs for pre-registration healthcare students – A review of the literature. Nurse Educ Today.

[19] Pelone F, Reeves S, Ioannides A, Emery C, Titmarsh K, Jackson M, Hassenkamp AM, Greenwood N (2015). Interprofessional education in the care of people diagnosed with dementia: protocol for a systematic review. BMJ Open.

[20] Walkenhorst U, Mahler C, Aistleithner R, Hahn EG, Kaap-Fröhlich S, Karstens S, Reiber K, Stock-Schröer B, Sottas B (2015). Position statement GMA Committee – "Interprofessional Education for the Health Care Professions". GMS Z Med Ausbild.

[21] Zumbach J, Spinath B, Schahn J, Friedrich M, Kögel M, Krämer M, Preiser S, Brusdeylins K (2007). Entwicklung einer Kurzskala zur Lehrevaluation. Psychologiedidaktik und Evaluation VI.

[22] Livingston G, Kelly L, Lewis-Holmes E, Baio G, Morris S, Patel N, Omar RZ, Katona C, Cooper C (2014). Non-pharmacological interventions for agitation in dementia: systematic review of randomised controlled trials. Br J Psychiatry.

[23] Spiers JA, Williams B, Gibson B, Kabotoff W, McIlwraith D, Sculley A, Richard E (2014). Graduate nurses' learning trajectories and experiences of problem based learning: a focused ethnography study. Int J Nurs Stud.

[24] Azer SA, Azer D (2015). Group interaction in problem-based learning tutorials: a systematic review. Eur J Dent Educ.

[25] Kong LN, Qin B, Zhou YQ, Mou SY, Gao HM (2014). The effectiveness of problem-based learning on development of nursing students' critical thinking: a systematic review and meta-analysis. Int J Nurs Stud.

[26] Onyon C (2012). Problem-based learning: a review of the educational and psychological theory. Clin Teach.

[27] Schmidt HG, Rotgans JI, Yew EH (2011). The process of problem-based learning: what works and why. Med Educ.

[28] Singler K, Stuck AE, Masud T, Goeldlin A, Roller RE (2014). Lernzielkatalog für die studentische Lehre im Fachbereich Geriatrie" an Fakultäten für Humanmedizin. Eine Empfehlung der Deutschen Gesellschaft für Geriatrie (DGG), der deutschen Gesellschaft für Gerontologie und Geriatrie (DGGG), der Österreichischen Gesellschaft für Geriatrie und Gerontologie (ÖGGG) und der Schweizerischen Fachgesellschaft für Geriatrie (SFGG) auf Basis der Empfehlungen der Europäischen Facharztvereinigung-Sektion Geriatrie (UEMS-GMS) 2013. Z Gerontol Geriatr.

[29] Traynor V, Inoue K, Crookes P (2011). Literature review: understanding nursing competence in dementia care. J Clin Nurs.

[30] Tsaroucha A, Benbow SM, Kingston P, Le Mesurier N (2013). Dementia skills for all: a core competency framework for the workforce in the United Kingdom. Dementia (London).

[31] George DR, Stuckey HL, Dillon CF, Whitehead MM (2011). Impact of participation in TimeSlips, a creative group-based storytelling program, on medical student attitudes toward persons with dementia: a qualitative study. Gerontologist.

[32] Isaacson RS, Safdieh JE, Ochner CN (2011). Effectiveness of a modified Continuum curriculum for medical students: a randomized trial. Neurology.

[33] Jefferson AL, Cantwell NG, Byerly LK, Morhardt D (2012). Medical student education program in Alzheimer's disease: the PAIRS Program. BMC Med Educ.

[34] Jonas-Simpson C, Mitchell GJ, Carson J, Whyte C, Dupuis S, Gillies J (2012). Phenomenological shifts for healthcare professionals after experiencing a research-based drama on living with dementia. J Adv Nurs.

[35] Paquette M, Bull M, Wilson S, Dreyfus L (2010). A complex elder care simulation using improvisational actors. Nurse Educ.

[36] Robinson A, Cubit K (2007). Caring for older people with dementia in residential care: nursing students' experiences. J Adv Nurs.

[37] Trail Ross ME (2012). Linking classroom learning to the community through service learning. J Community Health Nurs.

